# Traditional Chinese medicine on treating myelosuppression after chemotherapy: A protocol for systematic review and meta-analysis

**DOI:** 10.1097/MD.0000000000024307

**Published:** 2021-01-29

**Authors:** Sihang Yang, Hong Che, Li Xiao, Bingjie Zhao, Songshan Liu

**Affiliations:** Hospital of Chengdu University of Traditional Chinese Medicine, Chengdu, Sichuan, China.

**Keywords:** blood corpuscle, clinical symptoms, myelosuppression after chemotherapy, protocol, quality of life, systematic review, traditional Chinese medicine

## Abstract

**Background:**

Myelosuppression after chemotherapy is a common adverse reaction in the process of chemotherapy, mainly manifested as anemia, increased risk of bleeding, infection, the results seriously affect the quality of life and prognosis of patients, become the main cause of death. Since ancient times, traditional Chinese medicine (TCM) has been widely used in East Asia (such as China, Japan, South Korea) in the clinical treatment of bone marrow suppression after chemotherapy, which plays the role of synergism, toxicity reduction, immune regulation, and gradually developed into an indispensable role. Therefore, the purpose of this study was to use a network meta-analysis to evaluate the evidence that traditional Chinese medicine is related to the efficacy and safety of chemotherapy-induced myelosuppression.

**Methods:**

This study will search the following Chinese and English databases electronically: 4 Chinese literature databases, including China biology and medicine database, China National Knowledge Infrastructure, VIP, and Wan fang database, and 3 British literature databases including PubMed, EMBASE, and Cochrane Library. The search keywords were (traditional Chinese medicine or medicinal plants or extracts of traditional Chinese medicine or traditional Chinese medicine formula or preparation) and (myelosuppression after chemotherapy) and (randomized controlled trials) (RCTs). The search time limit is set to December 2020, and Chinese and English languages will be included. The included subjects must be diagnosed with myelosuppression after chemotherapy and RCTs should be conducted at the same time. The main outcome was elevated hemoglobin, platelets, leukocytes, and neutrophils. The secondary results were reticulocyte absolute value, reticulocyte percentage, low-fluorescence reticulocyte red, medium-fluorescent reticulocyte red, and high-fluorescence reticulocyte red. We will conduct a risk and quality assessment of the included studies using the Cochrane tool, and carefully calculate data synthesis after meta-analysis using Rev Man software (version 5.3.5) and R software (version 3.6.1).

**Results:**

The study is aim to evaluate the efficacy and safety of the treatment that traditional Chinese medicine for myelosuppression after chemotherapy.

**Conclusion:**

This study of the meta-analysis could provide evidence for clinicians and help patients to make a better choice.

**INPLASY registration number:**

INPLASY2020120097

## 1 Introduction

Malignant tumor has become a major disease endangering human health and life. In 2018, there were 18.1 million new cancer cases and 9.6 million cancer deaths worldwide. There are 3.804 million new cancer cases in China, accounting for 21.0% of the world's new cancer cases, and 2.296 million cancer deaths, accounting for 23.9% of global cancer deaths.^[1,2]^ Surgery is an important means of modern medical treatment of tumor, but even radical surgery, there are tumor cells remaining in the body. Chemotherapy, as a systematic treatment, is an important treatment for advanced tumors. Most of them use cytotoxic drugs to interfere with DNA and RNA synthesis of tumor cells or directly kill tumor cells to achieve the effect of antitumor treatment.^[3,4]^ Myelosuppression is the most common adverse reaction of chemotherapy, mainly manifested by the decrease of white blood cells, hemoglobin, platelet count, and bleeding. The severity and duration of neutropenia determine the risk of infection. Infection can rapidly lead to deterioration of clinical conditions. If the treatment is not appropriate, it will cause serious consequences.^[5–8]^ Some patients with malignant tumor may not die of disease soon, but may be fatal due to bone marrow suppression, which has become the main factor affecting the quality of life and survival time of patients. Modern medical treatment mainly includes transfusion of red blood cells and platelets and subcutaneous injection of stimulating factors. There are high treatment costs and many side effects of long-term use of drugs.^[9–11]^ Many patients have to seek other treatment methods to supplement and replace the above treatment.^[6,12]^

In recent years, traditional Chinese medicine (TCM) has been widely used in East Asia (such as China, Korea, and Japan) to treat patients with myelosuppression after chemotherapy, and has achieved good clinical efficacy. Sun and Wu^[13]^ found that the number of white blood cells, platelet count, and hemoglobin content in the experimental group were higher than those in the control group after chemotherapy. Jin et al^[14]^ research found that high dose of Qiao et al polysaccharide can accelerate the recovery of bone marrow hematopoietic function, reduce and prevent the toxic and side effects of chemotherapy. Liu^[15]^ used Dang gui Bu xue Decoction to increase the number of white blood cells and red blood cells in peripheral blood. Rehmannia glutinosa oligosaccharides and polysaccharides can promote the production and secretion of hematopoietic growth factors, which is conducive to bone marrow hematopoiesis.^[16]^ E jiao can promote hematopoietic function, increase the content of red blood cells and hemoglobin, stimulate the regeneration of platelets in peripheral blood, and increase white blood cells.^[17,18]^ There is no doubt that modern medical research has shown that traditional Chinese medicine has a more obvious therapeutic effect in strengthening and restoring bone marrow hematopoiesis. In addition, studies have also shown that a polysaccharide component of Angelica sinensis can not only promote the recovery of hematopoietic function of bone marrow, but also affect the production of 4 refined acid metabolites in vivo to prevent the progress of inflammation. In addition, it can stimulate the production of specific IgG antibodies.^[19]^ According to the basic theory of traditional Chinese medicine, we believe that Qi, blood, and body fluid are the driving force of human body and maintaining human life activities. The normal function of Qi, blood, and body fluid is closely related to kidney yin and kidney yang.^[20]^ “The spleen controls the blood, and the operation of the blood depends on the spleen” in the theory of blood syndrome. According to the theory of the origin of various diseases, “the kidney contains essence, and the essence is the result of blood.” The occurrence of hemocytopenia is closely related to the spleen and kidney. Therefore, the improvement of blood cell reduction should be started from the spleen and kidney.^[21,22]^ TCM has the advantages of low cost, good curative effect, convenient storage, easy to take, and so on.

Through the preliminary search of electronic database, we found that the randomized controlled trials (RCTs) of TCM in the treatment of post chemotherapy myelosuppression are increasing year by year. However, most clinical trials have small sample size, low quality, and lack of effective evidence-based exploration. The reason is that the size and quantity of clinical centers are limited. In addition, publications of similar system reviews cannot be retrieved in the database. Therefore, in order to provide the basis for clinical thinking, this paper will use meta-analysis to evaluate the efficacy and safety of traditional Chinese medicine in the treatment of myelosuppression after chemotherapy.

## 2 Methods

### 2.1 Objectives and registration

This article will assess the efficacy and safety of Traditional Chinese medicine on treating myelosuppression after chemotherapy. The protocol has been registered in International Platform of Registered Systematic Review and Meta-analysis Protocols (INPLASY) INPLASY2020120097. And the article will adhere to the Preferred Reporting Items for Systematic Reviews and Meta-Analyses Statement (PRISMA-P reporting guidelines).

### 2.2 Search strategy

The following Chinese and English databases were searched electronically by keyword combination mode: PubMed, EMBASE and Cochrane Library, 3 British literature databases including PubMed, EMBASE and Cochrane Library, and 4 Chinese literature databases, including Chinese national knowledge infrastructure, VIP and Wan fang database, such as myelosuppression after chemotherapy and traditional Chinese medicine or medicinal plants or extracts of traditional Chinese medicine or traditional Chinese medicine formula or Chinese medicine pharmaceutical preparations (TCM). We also limited the search time to December 2020 on the treatment of myelosuppression after chemotherapy with TCM. Table 1 provides the complete search strategy of PubMed. The search strategy of other electronic databases is similar to that of PubMed. The search terms in Chinese databases are the translation of the above words.

**Table 1:** The search strategy for PubMed.

Number	Search terms
#1	myelosuppression after chemotherapy [MeSH Terms]
#2	traditional Chinese medicine[Title/Abstract]OR Chinese medicine [Title/Abstract]OR Chinese herbal medicine [Title/Abstract]
#3	RCT[Title/Abstract]OR randomized controlled trial[Title/Abstract]
#4	Efficacy[Title/Abstract] OR Safety[Title/Abstract]
#5	#1and #2 and #3 and #4

### 2.3 Participant or population

All chemotherapy patients with myelosuppression after chemotherapy but not immediately life-threatening will be included. This shows that the participants in our study will not have any gender, age or race, region, and other restrictions.

### 2.4 Intervention

Choose TCM treatment that is not limited to dose, frequency, duration, or follow-up time (including traditional Chinese medicine extract, traditional Chinese medicine preparation, single or mixed Chinese medicine formula, Chinese medicine combined with 1 or more Western Medicine). TCM intravenous injection, acupuncture, massage, and other TCM treatment will be limited.

### 2.5 Comparator

For control intervention, patients who did not receive any treatment were regarded as blank control, and patients only treated by Western medicine were treated as control intervention. In addition, once patients use TCM during the trial, they should immediately refuse.

### 2.6 Study designs to be included

This study will collect all RCTs based studies of TCM in the treatment of postchemotherapy myelosuppression, whether they have been published or not. Quasi-RCTs, non-RCTs, case reports, and cross studies were not included in the collection.

### 2.7 Eligibility criteria

All chemotherapy patients with myelosuppression after chemotherapy but not immediately life-threatening will be included. This shows that the participants in our study will not have any gender, age or race, region, and other restrictions.

### 2.8 Information sources

The following Chinese and English databases were searched electronically by keyword combination mode: PubMed, EMBASE, and Cochrane Library, 3 British literature databases including PubMed, EMBASE, and Cochrane Library, and 4 Chinese literature databases, including Chinese national knowledge infrastructure, VIP, and Wan fang database, such as myelosuppression after chemotherapy and traditional Chinese medicine or medicinal plants or extracts of traditional Chinese medicine or traditional Chinese medicine formula or Chinese medicine pharmaceutical preparations (TCM). We also limited the search time to December 2020 on the treatment of myelosuppression after chemotherapy with TCM. We will also manually search for gray documents such as conference minutes, dissertations, and trial registration (through the WHO International Clinical Trial Registration Platform (ICRP) and the national registry website).

### 2.9 Types of outcome measures

#### 2.9.1 Primary outcomes

The main outcome was the elevation of hemoglobin, platelets, leukocytes, and neutrophils. The main outcome was the elevation of Hb and PLT and WBC.

#### 2.9.2 Secondary outcomes

- Clinical symptoms
- Quality of life
- Absolute value of reticulocyte (RTC)
- Percentage of RTC
- Low-fluorescent RTC
- Medium-fluorescent RTC
- High-fluorescent RTC

### 2.10 Study identification

**Figure 1:**
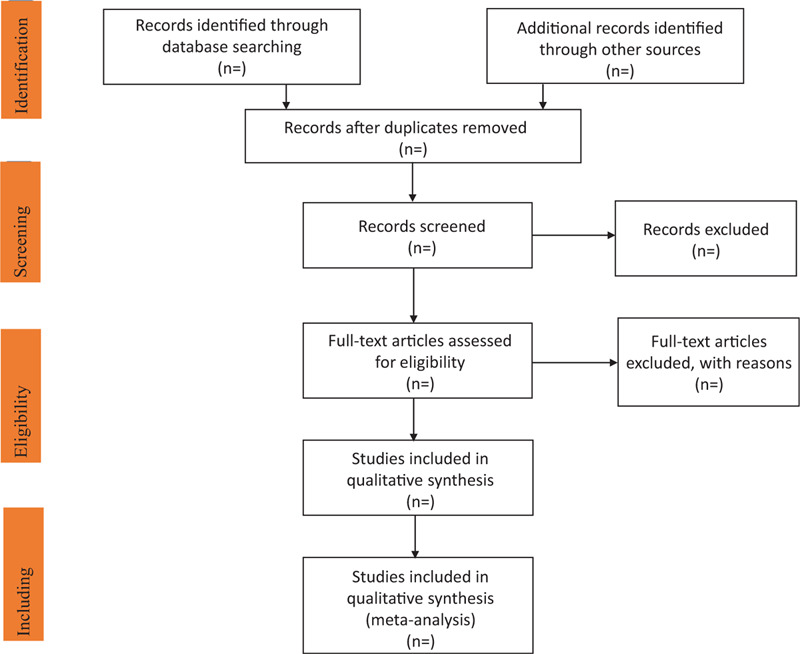
Flow chart of study selection.

First of all, the researchers will carefully discuss and determine the screening criteria within the group before conducting the study. Information retrieval and literature screening will be composed of 2 research members who are familiar with EndNoteX7 document management system and can quickly and effectively import all the documents into the system to delete unqualified documents. Then, we will exclude the obviously unqualified literature by reading the title and abstract. Then, we will browse the remaining literature to screen out the unqualified literature such as case report, theoretical analysis, and intervention. After that, we will carefully read the rest of the literature, strictly screen out the lack of control group, insufficient random distribution, and incompatible outcome indicators. Finally, after careful discussion by 2 research members, the literature that cannot be guaranteed will be handed over to another research member, which means that appropriate RCTs can be absorbed and involved in the research. A detailed summary of the study selection is shown in the PRISMA flowchart (Fig. 1).

### 2.11 Data management

Literature data collection will be input by 2 researchers through EpiData 3.1 software, and the final data collection will be updated to the database by another researcher after checking the consistency. The contents of data extraction include: title, first author, contact information, diagnostic criteria of myelosuppression, age, gender, severity, duration of suppression, intervention measures used in experimental and control groups, treatment time of TCM, safety indicators, and number of adverse reactions. If the research data is insufficient, unclear, or missing, we will contact the first author of the study through contact information to improve the information. Those who failed were excluded from the study.

### 2.12 Quality assessment/risk of bias analysis

The risk of bias in this study will be assessed from 7 perspectives of Cochrane tool: sequence generation, assignment concealment, blinding participants, blinding testers, blinding result assessors, incomplete result data, and selective reporting of results. The above assessment will be completed by 2 well-trained research members. If there is disagreement, the problem can be solved through group discussion. If the opinions are still different, actively contact the author or consult a third assessor. We will also assess the evidence quality of the main results. The assessment includes 5 aspects: heterogeneity, indirectness, bias risk, imprecision, and publication bias, and uses “low risk,” “high risk,” or “unclear risk of bias” to make corresponding judgments on each level of evidence. EPOC guidelines will be used to assess the risks of non-RCTs.

### 2.13 Strategy of data synthesis

Rev Man software (version 5.3.5) and R software (version 3.6.1) will be used for protocol of meta in this paper. If possible, the analysis of all results will be done by intention to treat. We will conduct an analysis to provide estimates of the effect of dichotomy data and continuous data with a confidence interval of 95%. For dichotomy data, we will use the risk ratio (RR) and the average difference (MD) of continuous data. We will explore heterogeneity before meta-analysis of the results. Standard chi-square test was used to detect the heterogeneity, and the significance level was *P* < 0.10. I^2^ statistics will be used to quantify the inconsistencies among studies and to assess the impact of heterogeneity on the meta-analysis. The results of dichotomy were obtained by mantel method, and the continuous results were obtained by de Simone and Laird inverse variance methods. Random effects models will be used to collect data.

### 2.14 Subgroup analysis

We will conduct subgroup and meta regression analysis. If enough studies are included (at least 10 trials), the aim is to explore the heterogeneity sources of myelosuppression in terms of classification, complications, Chinese medicine treatment course, and treatment plan.

### 2.15 Sensibility analysis

We will report the results of sensitivity analysis by eliminating low-quality tests to evaluate the reliability and robustness of the results.

### 2.16 Publication bias

When more than 10 studies are included in the meta-analysis, funnel plot and egger regression tests will be used to analyze whether there is publication bias. If necessary, we can also consult the research authors for more information to eliminate possible large probability reporting bias.

### 2.17 Evidence evaluation

We will assess the evidence quality of the main results through the proposed hierarchical assessment, development, and evaluation methods. The assessment includes 5 aspects: heterogeneity, indirectness, bias risk, imprecision, and publication bias, and uses “high,” “moderate,” “low,” and “very low” to make corresponding judgments on each level of evidence.

## 3 Discussion

With the accelerated pace of population aging, the number of patients diagnosed as malignant tumor is increasing year by year.^[23,24]^ Therefore, RCTs related to myelosuppression after chemotherapy are also booming.^[25,26]^ However, due to different factors such as the treatment of the disease and the degree of bone marrow suppression, it still cannot reach a satisfactory level, which is reflected in the fact that clinicians have not reached a consensus on the treatment principles and evaluation of the disease, and there is a lack of unified standardized standards. TCM has a positive role in promoting bone marrow hematopoiesis and restoring bone marrow hematopoietic function. In short, it has rich theoretical knowledge and clinical treatment experience in relieving myelosuppression after chemotherapy.^[27,28]^ Chinese medicine is an important part of TCM, which has the characteristics of low price, easy to use, easy to carry, and small side effects, which has been protecting human health for a long time. This therapy can achieve ideal therapeutic effect by regulating the immune system or tissue function of malignant tumor patients and maintaining the balance of yin and Yang.^[29,30]^ Although the specific mechanism of TCM treatment on chemotherapy-induced myelosuppression has not been fully studied, clinical studies have shown that TCM treatment of chemotherapy-induced myelosuppression can improve the symptoms of anemia, to a certain extent, it can also reduce the incidence of infection and avoid progressive aggravation of the disease.^[31]^ Through many inquiries, we know that the effectiveness of TCM in myelosuppression after chemotherapy has not been compared. Therefore, it is of great practical significance to use systematic review and meta-analysis to evaluate the efficacy and safety of TCM in the treatment of myelosuppression after chemotherapy. The results of this study can provide a possible ranking for the treatment of myelosuppression after chemotherapy with TCM. In addition, we hope that these results will provide clinicians with the basis for TCM treatment of postchemotherapy myelosuppression, and provide patients with the best choice of treatment by evaluating the evidence quality of the main results. In addition, although this study will conduct a comprehensive search, it will only search Chinese and English literature, not other languages, which may lead to some biases.

### 3.1 Ethics and dissemination

This paper systematically evaluates the efficacy and safety of TCM in the treatment of myelosuppression after chemotherapy, because this systematic review does not involve human beings and does not need ethical recognition. In addition, all data will be analyzed anonymously during the review process. We will publish this systematic review electronically in a peer-reviewed journal. The review of this system will provide medical practitioners with good practice guidance and information on the treatment of myelosuppression after chemotherapy with TCM.

## Author contributions

**Conceptualization:** Bingjie Zhao, Songshan Liu.

**Data curation:** Songshan Liu, Sihang Yang

**Formal analysis:** Sihang Yang, Hong Che, Li Xiao.

**Funding acquisition:** Hong Che.

**Methodology:** Sihang Yang, Li Xiao, Bingjie Zhao

**Project administration:** Sihang Yang, Hong Che, Bingjie Zhao

**Supervision:** Songshan Liu

**Writing – original draft:** Sihang Yang, Bingjie Zhao.

**Writing – review & editing:** Sihang Yang, Hong Che, Li Xiao

## Abbreviations

Abbreviations: Hb = hemoglobin, PLT= platelet, RCTs = randomized controlled trials, RTC= reticulocyte, TCM = traditional Chinese medicine, WBC = leukocyte.
